# Gefühlte Inflation als Bestimmungsgrund der Spar- und Konsumstruktur von Verbrauchern

**DOI:** 10.1007/s10273-022-3292-3

**Published:** 2022-10-20

**Authors:** Andreas Krämer, Daniel F. Heuermann, Thomas Burgartz

**Affiliations:** 1 exeo Strategic Consulting AG, Wittelsbacherring 24, 53115 Bonn, Deutschland; 2 Department for Business, Sport and Event Management & Psychology, University of Europe for Applied Sciences (Campus Berlin), Dessauer Str. 3-5, 10963 Berlin, Deutschland; 3 Fakultät Wirtschaft, University of Europe for Applied Sciences (Campus Iserlohn), Reiterweg 26B, 58636 Iserlohn, Deutschland

**Keywords:** E31, E21, D12

## Abstract

Die Inflationsrate hat im Euroraum zuletzt einen Höchstwert erreicht. Neben der amtlich gemessenen Inflationsrate wurde die subjektive Inflationswahrnehmung hingegen wenig beachtet. Dieser Beitrag füllt diese Lücke, indem auf Grundlage einer repräsentativen Befragung die subjektive Inflationswahrnehmung gemessen und diskutiert wird, welche Gründe zu einer Divergenz von gemessener und wahrgenommener Inflation führen. Weiterhin wird untersucht, welche Personengruppen sich besonders von steigenden Preisen betroffen fühlen und wie Haushalte ihr Konsum- und Sparverhalten an die subjektive Inflationswahrnehmung anpassen.

## Abstract (en)

In Europe and the U. S., official inflation rates reached a level of around 7 %–8 % in mid-2022. Based on survey data for Germany, we show that there is a rising gap between the objective (administrative) inflation rate and the (subjective) inflation rate as perceived by consumers, which is around 13 %. Low-income households are particularly affected by rising prices. Across all income groups, consumers react to rising prices by means of purchasing and consumption restraints, which runs counter to an assumed preference for the present as postulated by classical macroeconomics.

Die Teuerungsrate im Euroraum hat im Juni 2022 einen Wert von 8,6 % und damit den höchsten Wert in der Geschichte des Euro erreicht (EZB, 2022). Auch in den USA sind die Preise im April im Vergleich zum Vorjahr um 8,4 % gestiegen (Trading Economics, 2022). DeSilver (2022) zeigt anhand internationaler Daten, dass hohe Inflationsraten im Jahr 2022 zu einem weltweiten Phänomen geworden sind. Ihren Anfang nahmen diese historisch gesehen überdurchschnittlichen Werte im Sommer 2021, als die Inflation in den USA und im Euroraum die Dreiprozentmarke überschritt. Seitdem hat das Thema steigender Preise zunehmend den politischen und gesellschaftlichen Diskurs erreicht. So nehmen derzeit 70 % der US-Amerikaner:innen die Inflation als „sehr großes Problem“ wahr (Pew Research Center, 2022). Ähnlich stellt sich die Situation in Deutschland dar, wo 37 % der Menschen bereits im September 2021 steigende Preise als eines der drei größten gegenwärtigen Probleme erachteten (IPSOS, 2021); im Mai 2022 sahen 40 % der Menschen hier die Inflation als das größte gesellschaftliche Problem an (McKinsey, 2022a). Im Juni 2022 ist dieser Wert weiter auf 48 % angestiegen (McKinsey, 2022b). Laut Umfragen der Deutschen Bundesbank (2022) rechnen etwa 84 % der Deutschen mit einem weiteren Anstieg der Inflationsrate. Mit dem erwarteten Anstieg der Teuerungsrate und der zunehmend hohen Besorgnis in der Bevölkerung steht Deutschland nicht alleine da, vielmehr reflektiert dies einen weltweiten Trend (IPSOS, 2022).

**Prof. Dr. Andreas Krämer** ist Vorstandsvorsitzender der exeo Strategic Consulting AG in Bonn und Direktor des VARI e. V. (Value Research Institute) in Iserlohn.

**Prof. Dr. Daniel F. Heuermann** ist Professor für Wirtschaftswissenschaften an der University of Europe for Applied Sciences in Berlin.

**Prof. Dr. Thomas Burgartz** ist Professor für Performance Measurement an der University of Europe for Applied Sciences in Iserlohn und Direktor des VARI e. V. (Value Research Institute).

Anders als die amtlich gemessenen Inflationsraten hat die subjektive Inflationswahrnehmung der Menschen trotz der steigenden Bedeutung des Themas relativ wenig Beachtung erfahren. Zwar ist aus Studien bekannt, dass die Steigerung der Preise subjektiv zumeist als höher empfunden wird als von der amtlichen Statistik ausgewiesen (Arioli et al., 2017; Abildgren und Kuchler, 2019; Meyler und Reiche, 2021). Entsprechende Zusammenhänge wurden bereits unmittelbar nach Einführung des Euro diskutiert, als hohe wahrgenommene Preissteigerungen im Kontrast zu vergleichsweise geringen amtlichen Teuerungsraten standen (Aalto-Setälä, 2006). Gleichzeitig liegt jedoch keine belastbare Evidenz vor, ob und wie sich diese Wahrnehmung gerade in der gegenwärtigen Situation stark steigender Preise verändert hat. In diesem Kontext widmet sich der Beitrag den folgenden drei Kernfragen: In welchem Maße weichen in der aktuellen Situation die objektive und die subjektive Inflation voneinander ab? Wie lässt sich eine solche Divergenz erklären? Welche Personengruppen sind in besonderem Maße von einer steigenden Inflation betroffen und welche Verhaltensanpassun-

Abbildung 1

**Gefühlte Veränderung der Verbraucherpreise in den vergangenen 12 Monaten**

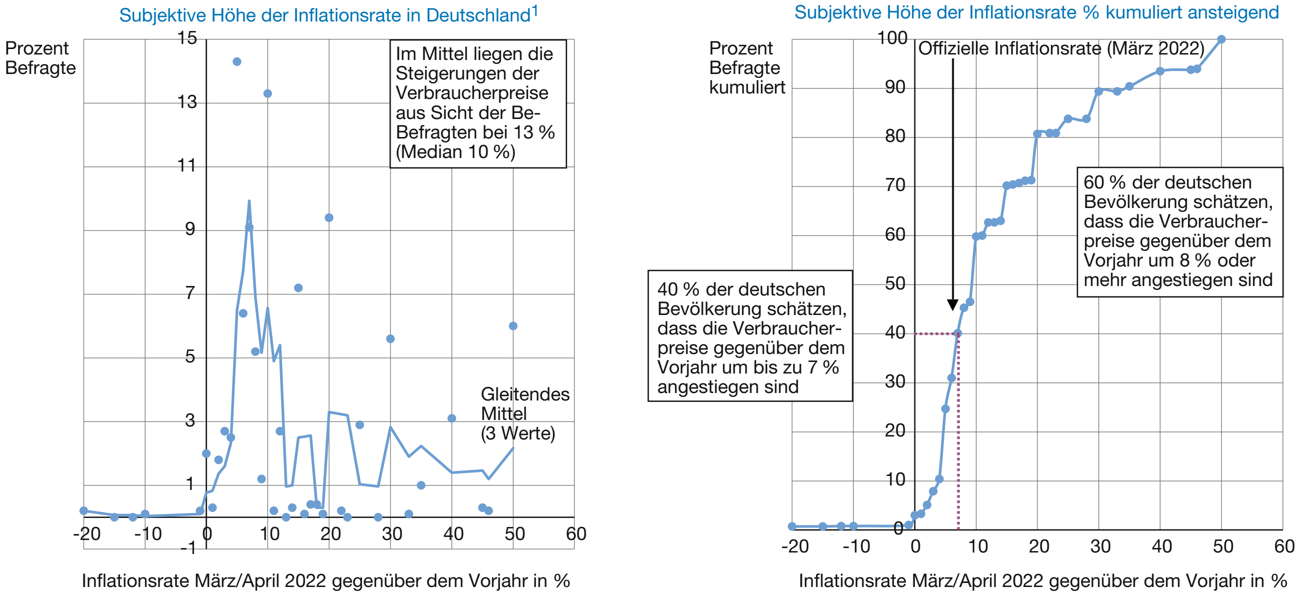

[1] Wie haben sich Ihrer Meinung nach die Verbraucherpreise in den letzten 12 Monaten entwickelt? Im Vergleich zum letzten Jahr sind diese aktuell… Antwortmöglichkeiten von „Die Preise sind stark gestiegen“ bis „Die Preise sind gesunken“. Und: Um wie viel Prozent sind Ihrer Meinung nach die Verbraucherpreise in den letzten 12 Monaten gestiegen / gesunken?

Quelle: exeo Strategic Consulting AG / Rogator AG.

gen nehmen diese vor? Beantwortet werden die Fragen auf der Basis einer aktuellen empirischen Untersuchung. Die Ergebnisse hieraus werden im Sinne eines Multi-Source-Multi-Method-Ansatzes mit anderen Datenquellen verzahnt.

## Daten und Methodik

„OpinionTRAIN“ ist eine repräsentativ angelegte Studie zur Bewertung von Trends und des Wertewandels in der Bevölkerung. Grundlage der Untersuchung ist eine Online-Befragung von Personen (18 bis 80 Jahre) u. a. in Deutschland. Seit Beginn der Coronapandemie wurden insgesamt vier Messungen zu verschiedenen Zeitpunkten durchgeführt. Im August/September 2021 wurde die dritte Erhebung durchgeführt, nachdem die erste Erhebung (April/Mai 2020) die Situation der Menschen zur Zeit des ersten Lockdowns beleuchtete und die zweite Erhebung (November/Dezember 2020) den Fokus auf den zweiten Lockdown legte. Die vierte Erhebung im März/April 2022 umfasst eine Befragung von 1.357 Personen, die nur in Deutschland durchgeführt wurde. Die Rekrutierung zur Erhebung erfolgte auf Basis von Online Access Panels. Um die Repräsentativität der Daten sicherzustellen, wurden die Daten in einem mehrstufigen Prozess gewichtet. In allen vier Erhebungswellen wurden Fragen zur finanziellen Situation (Sparneigung, Geldanlage, Kenntnis der offiziellen Inflationsrate, etc.) gestellt, sodass auf dieser Grundlage Zeitreihenanalysen möglich sind. In der jüngsten Erhebung wurden die Studienteilnehmenden zusätzlich um ihre Einschätzung der allgemeinen Teuerungsrate gebeten.

## Subjektive und objektive Inflationsraten

Verschiedene Studien haben in der Vergangenheit gezeigt, dass Konsument:innen Preissteigerungen üblicherweise als höher empfinden als von der amtlichen Statistik gemessen. Diese Erkenntnis ist über die Zeit hinweg stabil, jedoch unterscheidet sich die subjektive Inflationswahrnehmung zwischen Ländern und Personengruppen. So zeigen Arioli et al. (2017) für den Euroraum, dass die subjektive Inflationsrate die amtlichen Werte im Mittel um 4 % übersteigt, wobei dieser Abstand im südlichen Europa größer ist als in den nördlichen Ländern. Meyler und Reiche (2021) ergänzen diese Erkenntnisse dahingehend, dass der Abstand zwischen subjektiver und objektiver Inflation mit dem Alter, dem Bildungsstand sowie dem Haushaltseinkommen abnimmt. Aktuelle Daten, die diese Zusammenhänge in der derzeitigen Phase hoher allgemeiner Preissteigerungen beleuchten, liegen jedoch bisher nicht vor.

In einem ersten Schritt wird vor diesem Hintergrund die subjektive Inflationswahrnehmung in Deutschland untersucht. Abbildung 1 stellt die Ergebnisse für den Monat April 2022 grafisch dar. Die linke Seite zeigt die Verteilung der Antwor-

Abbildung 2

**Beurteilung der Veränderungen der Preise und Ausgaben der Verbraucher:innen bei Lebensmitteln**

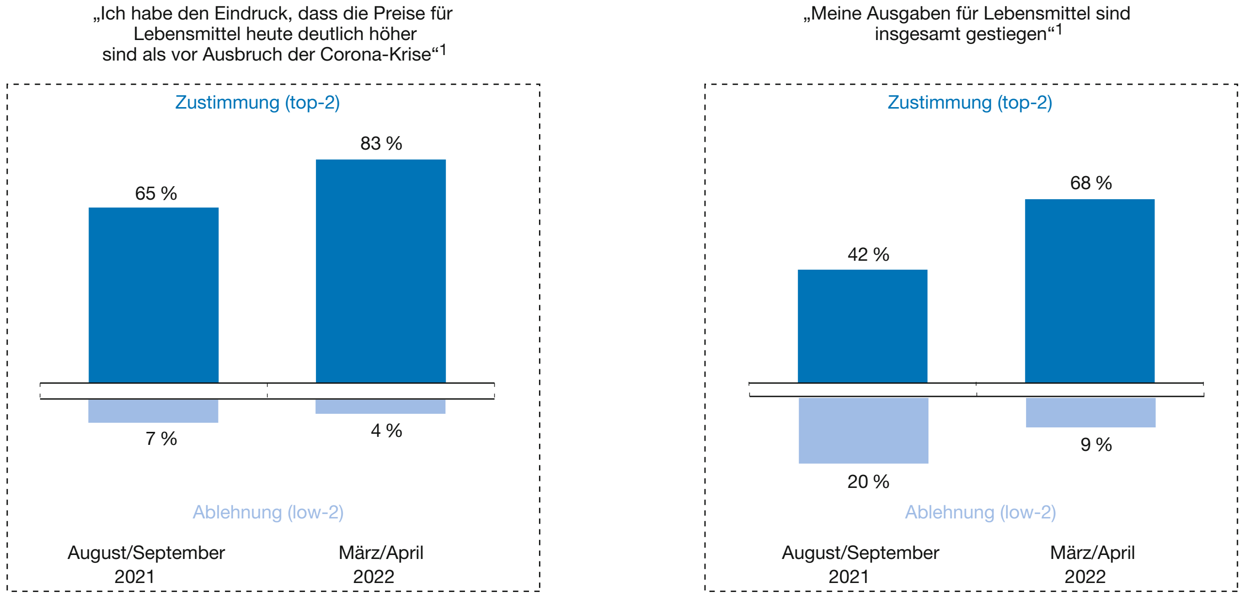

[1] Wir kommen jetzt zum Thema Einkaufsverhalten: Bitte bewerten Sie die folgenden Aussagen zum Einkauf in der aktuellen Zeit der Coronakrise mit Werten von 1 = „stimme sehr zu“ bis 6 = „stimme gar nicht zu“. Top-2 = Skalenpunkte 1 und 2; low-2 = Skalenpunkte 5 und 6.

Quelle: exeo Strategic Consulting AG / Rogator AG.

ten. Auffällig ist zunächst die Häufung der Werte an salienten Punkten, insbesondere an Vielfachen von fünf. Besonders häufig werden subjektive Inflationsraten von 5 %, 10 %, 15 % und 20 % genannt. Der Mittelwert der Antworten liegt bei 13 %, der Median bei 10 %. Dieser Wert liegt deutlich oberhalb der subjektiven Inflation von 4,5 %, die Schnabl und Sepp (2021) sowie die UniCredit Bank für das erste Quartal 2021 ermittelt haben, und ebenfalls höher als der Wert von 8,5 %, den Schnabl (2022) für das erste Quartal 2022 auf Grundlage einer linearen Fortschreibung früherer subjektiver Inflationsraten geschätzt hat. Die amtlich gemessene Inflationsrate lag zum Zeitpunkt der Befragung bei 7,4 %. Die rechte Seite zeigt die Verteilung der Schätzwerte kumuliert aufsteigend. Deutlich wird, dass lediglich jeder Vierte die Inflationsrate bei bis zu 5 % einschätzt. 60 % der Befragten geben eine subjektive Rate oberhalb des amtlichen Wertes an, wobei nahezu ein Drittel der Antworten sogar oberhalb von 15 % liegen. Zusammenfassend lässt sich sagen, dass konsistent mit der bestehenden Literatur auch die Mehrheit der Konsument:innen in Deutschland die Teuerungsrate als höher empfindet, als diese in der amtlichen Statistik ausgewiesen wird.

## Unterschiede zwischen objektiver und subjektiver Inflation

Die beobachtete Divergenz der subjektiven und der objektiven Inflationsrate ist grundsätzlich konsistent mit der aus der Verhaltensökonomie bekannten Verlustaversion von Individuen. Der Begriff Verlustaversion beschreibt die empirische Beobachtung, dass Menschen üblicherweise Verlusten eine größere Bedeutung beimessen als Gewinnen (Kahneman und Tversky, 1992). Im vorliegenden Kontext bedeutet dies, dass Preissteigerungen möglicherweise subjektiv stärker wahrgenommen werden als Preissenkungen. Diese kann insbesondere dann zum Tragen kommen, wenn gerade Güter des täglichen Bedarfs überproportional teurer werden (Dreger, 2008).

Bereits im Nachgang zur Euro-Umstellung wurden Hypothesen zur Inflationswahrnehmung aufgestellt (Brachinger, 2005; Fluch et al., 2005), nach denen Preisanstiege von Konsument:innen stärker registriert werden als Preissenkungen. Um zu ermitteln, wie stark unterschiedliche Bevölkerungsgruppen von der Inflation betroffen sind, schlägt Brachinger (2008) vor, „zwei Teuerungsraten zu ermitteln: eine Rate zur Erfassung der Teuerung bei den Gütern des täglichen Konsums und eine zweite zur Erfassung der Teuerung bei den langlebigen Gebrauchsgütern. Die gesamte Teuerung ergibt sich dann als gewogenes Mittel der beiden Teilraten.“ Ein solcher Vorschlag steht in inhaltlichem Zusammenhang mit der Frage, ob die offizielle Teuerungsrate (in Zeiten unter anderem durch die Coronapandemie stark veränderter Konsumstrukturen) nach wie vor auf einem repräsentativen Warenkorb basiert. So zeigt z. B. Cavallo (2020), dass die Inflationsrate in den USA bei Covid-angepassten Warenkörben höher als die offiziell ausgewie-

Abbildung 3

**Überproportional hohe wahrgenommene Inflation nach Teilsegmenten**

Einschätzung zur Entwicklung der Verbraucherpreise in den letzten 12 Monaten[1]

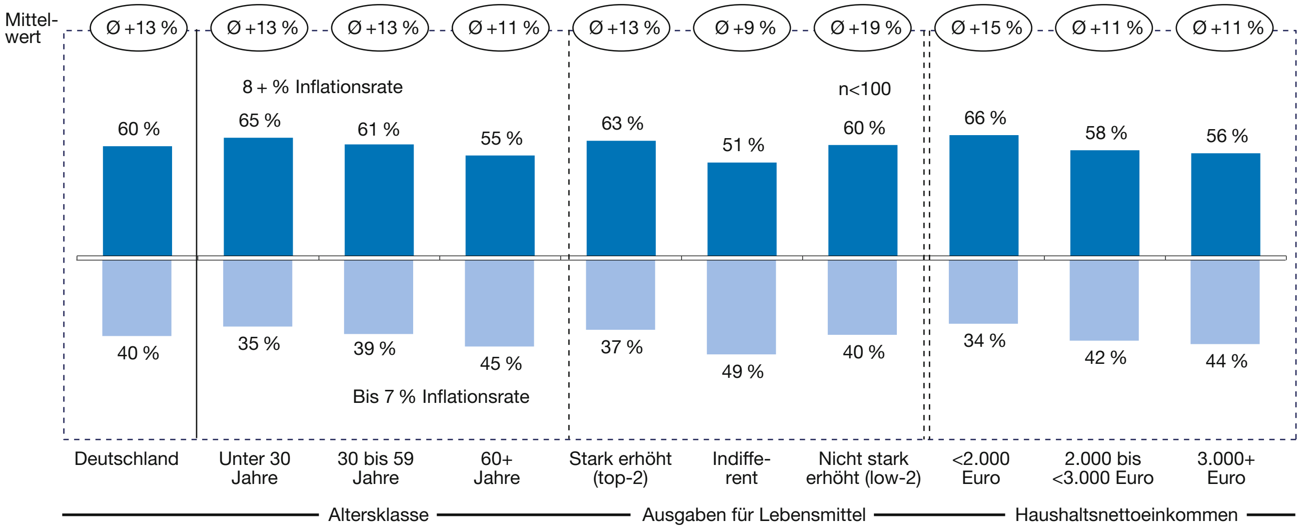

[1] Wie haben sich Ihrer Meinung nach die Verbraucherpreise in den letzten 12 Monaten entwickelt? Im Vergleich zum letzten Jahr sind diese aktuell… Antwortmöglichkeiten von „Die Preise sind stark gestiegen" bis „Die Preise sind gesunken". Und: Um wie viel Prozent sind Ihrer Meinung nach die Verbraucherpreise in den letzten 12 Monaten gestiegen / gesunken? Oben als Balken dargestellt ist der Anteil der Personen innerhalb der Klasse, die die Inflation bei 8 % und höher sehen.

Quelle: exeo Strategic Consulting AG / Rogator AG.

sene Inflationsrate ist. Eine solche überdurchschnittliche Teuerung von Produkten des täglichen Bedarfs ist auch in Deutschland zu beobachten. So zeigt Kecskes (2022), dass die Preise der Eigenmarken der Discounter deutlich mehr als die durchschnittliche Inflationsrate gestiegen sind.

Diese Entwicklung spiegelt sich in der Wahrnehmung der Konsument:innen wider. Abbildung 2 zeigt, dass der Anteil der Befragten, die angeben, dass die Preise zum Befragungszeitpunkt deutlich höher liegen als vor Beginn der Coronapandemie, von 65 % im August/September 2021 auf 83 % im März/April 2022 gestiegen ist. In der Konsequenz ist auch der Anteil derjenigen Haushalte, die mehr Geld für Lebensmittel ausgeben als vor der Pandemie, um mehr als 50 % gestiegen. Für die weitere Diskussion ist zudem von Interesse, dass offensichtlich die Preissteigerungen stärker wahrgenommen werden als wachsende Ausgaben für Lebensmittel. Vermutet werden kann, dass hier Anpassungen in der Konsumstruktur zum Ausdruck kommen.

Eine differenzierte Betrachtung einzelner Konsumentengruppen erlaubt es, den Zusammenhang zwischen subjektiver Inflationsrate und Konsumverhalten näher zu beleuchten. In Abbildung 3 sind hierzu neben einer Einteilung nach Alters- oder Einkommensgruppen die Konsument:innen gemäß ihrer Ausgabensteigerung bei Lebensmitteln klassifiziert. Deutlich wird, dass sich die subjektive Inflationswahrnehmung zwischen denjenigen Haushalten, deren Ausgaben für Lebensmitteln stark gestiegen sind, nicht signifikant von denjenigen unterscheidet, deren Ausgaben mehr oder weniger konstant geblieben sind. Dies spricht dagegen, dass die subjektive Inflationsrate in der aktuellen Situation primär von Preissteigerungen bei Gütern des täglichen Bedarfs getrieben ist.

Unabhängig davon zeigt sich eine Tendenz hin zur Substitution von teureren durch günstigere Güter. So ist laut Kecskes (2022) der Umsatzanteil der Eigenmarken erstmalig seit langem wieder angestiegen und erreichte im ersten Quartal einen Wert von 34,6 %. Auch andere Studien deuten auf eine steigende Preissensitivität im Zuge erhöhter Inflationsraten hin. Bei den relevanten Entscheidungskriterien rückt der Preis aktuell stärker in den Vordergrund (idealo.de, 2022).

## Betroffenheit der Bevölkerungsgruppen

Hinsichtlich der Verteilungswirkung stellt sich die Frage, welche Teile der Bevölkerung in besonderem Maße von steigenden Inflationsraten betroffen sind. Diese Frage lässt sich aus zwei Blickwinkeln beantworten. Zum einen erlaubt eine Differenzierung nach Einkommensgruppen Einblicke in mögliche Unterschiede hinsichtlich der subjektiven Betroffenheit von Preissteigerungen. Alternativ hierzu können „offenbarte Präferenzen" (*revealed preferences*), d. h. durch konkrete Handlungen zum Ausdruck gebrachte Entscheidungsmuster, herangezogen werden. Im vorliegenden

Abbildung 4

## Aussagen zur Spar- und Konsumneigung nach Haushaltseinkommen

März/April 2022: Aussagen zu den Themen Sparen, Zinsen und Inflation nach subjektiver Inflationsrate[1]

| Zustimmung zum Statement (% der Befragten) | < 2.000 Euro HHNE | April/Mai 2020 | 2.000 - < 3.000 Euro HHNE | April/Mai 2020 | 3.000+ Euro HHNE | April/Mai 2020 |
|---|---|---|---|---|---|---|
| Ich schaffe es, jeden Monat etwas Geld zu sparen | 28 % | 32 % | 44 % | 51 % | 55 % | 66 % |
| Ich plane, in den nächsten Jahren einen größeren Kredit aufzunehmen (z. B. für einen Hauskauf) | 4 % | 3 % | 6 % | 8 % | 10 % | 5 % |
| Ich bin besorgt über die niedrigen Zinsen, die ich auf der Bank für mein Guthaben bekomme | 17 % | 18 % | 24 % | 30 % | 24 % | 25 % |
| Ich nehme stärker Kredite auf, weil die Zinsen so günstig sind | 2 % | 3 % | 4 % | 6 % | 2 % | 5 % |
| Ich werde zukünftig mehr Geld für Konsum ausgeben, weil sich Geld-Sparen nicht mehr lohnt | 13 % | 8 % | 9 % | 16 % | 8 % | 11% |
| Ich kenne die aktuelle Inflationsrate (allgemeine Preissteigerungsrate) des Statistischen Bundesamtes | 18 % | 7 % | 23 % | 11 % | 28 % | 18 % |
| Ich habe das Gefühl, dass die Preise, die ich persönlich zahle, stärker steigen als die amtliche Inflationsrate | 49 % | 34 % | 39 % | 34 % | 43 % | 23 % |
| Keine dieser Aussagen trifft zu | 13 % | 25 % | 10 % | 13 % | 8 % | 11 % |

[1] Wenn Sie einmal an Ihre Finanzsituation denken, welche Aussagen treffen dann für Sie zu?

Quelle: exeo Strategic Consulting AG / Rogator AG.

Kontext verspricht z. B. eine Analyse der Sparquoten nach Haushaltstypen einen Einblick in Unterschiede bei der Betroffenheit von Inflation. So ließe sich eine überproportionale Abnahme der Sparquote bei Geringverdienenden als Indiz dafür interpretieren, dass gerade in dieser Gruppe ein zunehmender Anteil des Einkommens für die Aufrechterhaltung des Lebensstandards eingesetzt werden muss.

Abbildung 3 stellt ebenfalls die Einschätzung der Teuerungsrate getrennt nach Einkommensgruppen dar. Deutlich wird, dass die subjektiv wahrgenommene Inflation mit steigendem Haushaltseinkommen abnimmt. So geben 66 % der Haushalte mit einem Nettoeinkommen von weniger als 2.000 Euro eine subjektive Inflationsrate von mindestens 8 % an, während dieser Anteil bei Haushalten mit einem Nettoeinkommen von mehr als 3.000 Euro nur bei 56 % liegt. Dies ist konsistent mit dem Argument, dass Bezieher:innen von niedrigen Einkommen sich in stärkerem Maße von Preissteigerungen betroffen fühlen als Personen mit hohem Einkommen.

Abbildung 4 stellt in der ersten Zeile den Anteil der Haushalte dar, die nach eigenen Angaben in der Lage sind, jeden Monat etwas Geld zu sparen. Erwartungsgemäß nimmt dieser Anteil mit steigendem Einkommen zu. So sparen derzeit lediglich 28 % der Haushalte mit einem Nettoeinkommen von weniger als 2.000 monatlich einen gewissen Betrag; bei Haushalten mit einem Einkommen von mehr als 3.000 Euro beträgt dieser Anteil 55 %. Die Abbildung ermöglicht darüber hinaus durch einen Vergleich mit den korrespondierenden Ergebnissen aus der Untersuchungswelle April/Mai 2020 einen Einblick in die Veränderung dieser Werte über die Zeit. Insgesamt zeigt sich ein deutlich dämpfender Effekt, d. h. der Anteil der Befragten, die angeben, etwas Geld sparen zu können, sinkt in allen Einkommensklassen.

Einwenden ließe sich hier, dass die Sparquote 2021 pandemiebedingt mit gut 16 % auf dem höchsten Niveau seit der deutschen Wiedervereinigung lag (Statista, 2022). Entsprechend wäre es denkbar, dass ein Teil des Rückgangs der sparenden Haushalte durch Nachholeffekte beim privaten Konsum zu erklären ist. Dies wäre grundsätzlich konsistent mit dem theoretischen Konzept einer Gegenwartspräferenz im Angesicht steigender Preise. Diese Erklärung für das sinkende Sparverhalten steht jedoch in deutlichem Widerspruch zu den Ergebnissen des Consumer Sentiment Survey von McKinsey (2022b), der zeigt, dass nahezu die Hälfte aller Konsument:innen angesichts steigender Preise ihr Konsumverhalten bereits eingeschränkt haben oder dies zu tun gedenken. Dies wird durch die jüngsten Ergebnisse der Gesellschaft für Konsumforschung bestätigt, die bereits jetzt einen deutlichen Rückgang der Konsumneigung feststellt (GfK, 2022). Hinsichtlich der Möglichkeit des Sparens führte der Präsident des Sparkassenverbands Baden-Württemberg im August 2022 aus, dass „mittlerweile rund 40 % unserer Privatkundinnen und -kunden kein Geld zur Seite legen können" und dies eine „dramatische Zahl" sei (Focus, 2022a).

Wenngleich sich die Ursachen einer sinkenden Sparneigung auf Grundlage der Daten nicht abschließend klären lassen, zeigt sich, dass Haushalte mit geringem Einkommen die Inflation stärker wahrnehmen als Haushalte mit höherem Einkommen und gleichzeitig die Möglichkeit des

Abbildung 5
**Zeitreihen ausgewählter Parameter zur Inflation und Sparneigung**

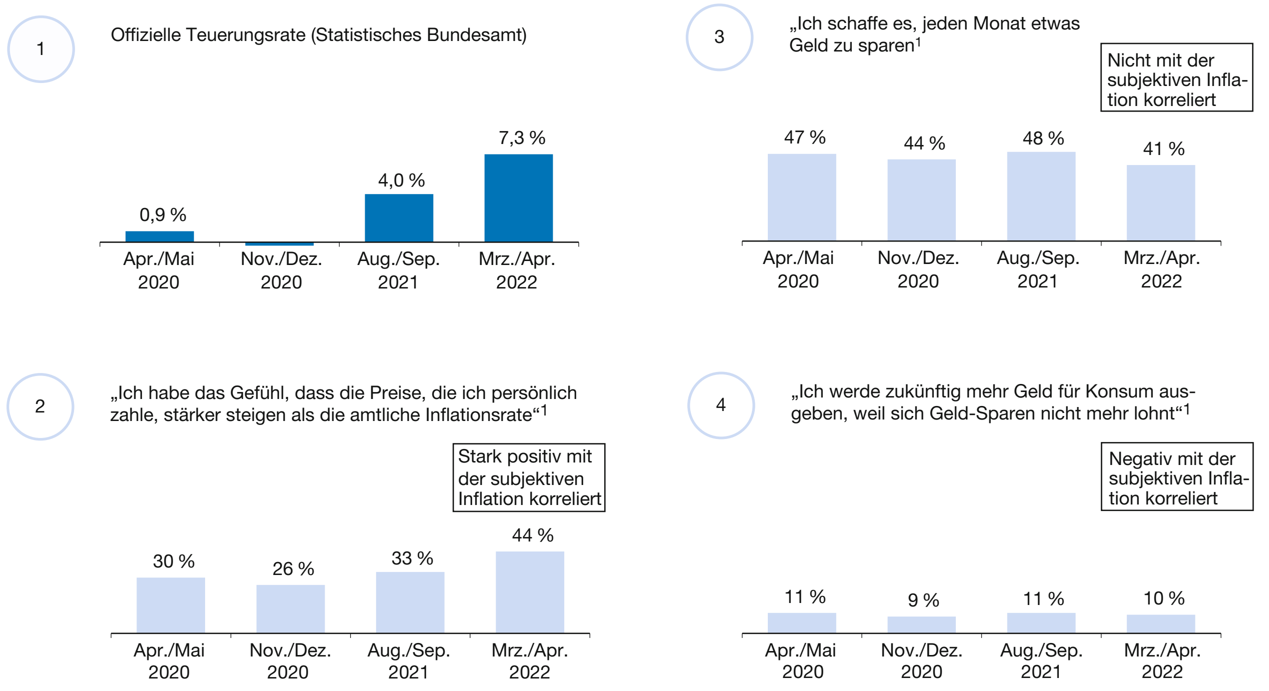

[1] Wenn Sie einmal an Ihre Finanzsituation denken, welche Aussagen treffen dann für Sie zu?

Quelle: exeo Strategic Consulting AG / Rogator AG.

Sparens quer durch alle Einkommensgruppen zwischen vier und 11 Prozentpunkten abnimmt.

## Zusammenfassung und Ausblick

Abbildung 5 fasst die wesentlichen Ergebnisse der Studie zusammen. Zunächst zeigt sich, dass der Anteil der Konsument:innen, die Preise als stärker steigend empfinden als von der amtlichen Statistik ausgewiesen, mit höherer Inflation zunimmt. Dies bedeutet, dass die subjektive Inflationswahrnehmung nicht linear mit der amtlich gemessenen Preissteigerung variiert, sondern überproportional zunimmt (Teilabbildung 1 und 2). Gerade in Zeiten hoher Inflation nehmen Menschen diese als noch ausgeprägter wahr, als sie offiziell gemessen wird. Weiterhin lassen die Ergebnisse die Schlussfolgerung zu, dass sich die höhere Inflationswahrnehmung quer durch alle Gesellschaftsgruppen zieht, ökonomisch schwächere Gruppen jedoch stärker von Preissteigerungen betroffen sind. Dies zeigt sich zum einen an einer ausgeprägteren Wahrnehmung der Inflation und zum anderen durch eine geringere Sparneigung (Teilabbildung 3). Gleichzeitig schlägt sich die geringere Sparquote nicht in einem höheren Konsumniveau nieder. Dies ist eine wichtige Erkenntnis, da das Ergebnis konträr zu den Annahmen in der Makroökonmie ist. Theoretische Standardmodelle sagen voraus, dass höhere Inflationserwartungen an der Nullzinsgrenze zu einem stärkeren Anstieg der Verbraucherausgaben führen (Ichiue und Nishiguchi, 2015). Ein stärkerer Konsum wird selbst dann angenommen, wenn der Realzins steigt (Rygaert, 2022). Allerdings liegen auch Studienergebnisse vor, die eine Einschränkung des Konsums durch eine allgemein starke Unsicherheit bestimmt sehen (Armantier et al., 2021).

Wie ebenfalls in Abbildung 5 zu sehen ist, bleibt der Anteil derjenigen Personen, die planen, mehr Geld für Konsum auszugeben, über die Zeit mit 9 % bis 11 % bemerkenswert stabil (Teilabbildung 4). Entgegen der makroökonomischen Theorie, die einen stärkeren Konsum unterstellt, wenn Geld seine Kaufkraft verliert, deuten die Studienergebnisse auf unveränderte Konsumneigungen bei gleichzeitig stark erhöhter Inflation. Zu vermuten ist jedoch, dass dies in eine Gegenbewegung umschlagen kann, wenn weitergehende Angebotsverknappungen auf steigende Preiserwartungen treffen (von Wallwitz, 2022; Schieritz, 2020).

Sowohl das Gefühl der individuellen Betroffenheit, welches auch in der Wochenzeitung Die Zeit (2022) im Rahmen von Einzelinterviews dokumentiert wird, als auch die sozialen Verteilungseffekte zeugen von der erheblichen sozialen und politischen Sprengkraft, den die derzeitig historisch hohen Inflationsraten bergen. Und auch auf der Seite der Unternehmen sorgen anhaltende handelspolitische Konflikte sowie gravierende Versorgungsrisiken für deutliche Anpassungen in der Geschäftsstrategie und Preissetzung. So ist zu beobachten, dass sich viele Unternehmen aufgrund der neu entstandenen Rahmenbedingungen in Krisenzeiten mit der Etablierung eines risikoorientierten Managements beschäftigen, was auch ein kundenorientiertes Risikomanagement einschließt (Burgartz und Krämer, 2020). Auch Preissteigerungen als Reaktion auf die Inflation sind bei zahlreichen Unternehmen aktuell zu beobachten. So trifft die Inflation etwa auch die Möbelbranche; als prominentes Beispiel hat IKEA in diesem Jahr die Preise deutlich stärker erhöht als die Inflation. Dies ist kein Einzelfall. Hauptursache für die kräftigen Preisanhebungen sind gestiegene Kosten bei der Beschaffung von Energie, Rohstoffen, sonstigen Vorprodukten und Handelswaren (Focus, 2022b). Angesichts der Tatsache, dass ein Teil der Inflation auf Angebotsschocks als Ergebnis unterbrochener und unsicherer Lieferketten zurückzuführen ist, können Zinssteigerungen nur einen Teil der politischen Maßnahmen darstellen. Gleichzeitig dürfte es mit Blick auf die in dieser Studie vorgelegten Ergebnisse von hoher Priorität sein, insbesondere die sozialen und gesellschaftlichen Verteilungseffekte zumindest kurzfristig zielgruppenspezifisch über steuer- oder fiskalpolitische Maßnahmen abzufedern.
